# High-pressure torsion for new hydrogen storage materials

**DOI:** 10.1080/14686996.2018.1435131

**Published:** 2018-02-19

**Authors:** Kaveh Edalati, Etsuo Akiba, Zenji Horita

**Affiliations:** ^a^ WPI, International Institute for Carbon-Neutral Energy Research (WPI-I2CNER), Kyushu University, Fukuoka, Japan; ^b^ Faculty of Engineering, Department of Materials Science and Engineering, Kyushu University, Fukuoka, Japan; ^c^ Faculty of Engineering, Department of Mechanical Engineering, Kyushu University, Fukuoka, Japan

**Keywords:** Solid-state hydrogen storage, metal hydrides, carbon-neutral energy, severe plastic deformation (SPD), nanostructured materials, ultrafine grain (UFG), lattice defects, grain boundaries, Mg-based alloys, Ti-based intermetallics, 50 Energy Materials, 206 Energy conversion / transport / storage / recovery

## Abstract

High-pressure torsion (HPT) is widely used as a severe plastic deformation technique to create ultrafine-grained structures with promising mechanical and functional properties. Since 2007, the method has been employed to enhance the hydrogenation kinetics in different Mg-based hydrogen storage materials. Recent studies showed that the method is effective not only for increasing the hydrogenation kinetics but also for improving the hydrogenation activity, for enhancing the air resistivity and more importantly for synthesizing new nanostructured hydrogen storage materials with high densities of lattice defects. This manuscript reviews some major findings on the impact of HPT process on the hydrogen storage performance of different titanium-based and magnesium-based materials.

## Graphical abstract

**Figure:**
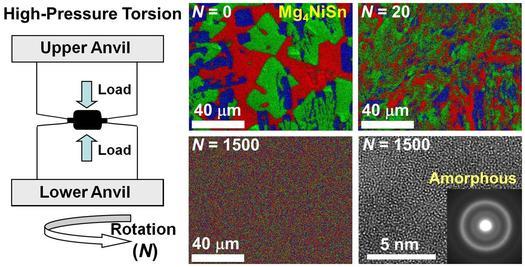

## 1. Introduction

**Figure 1.**
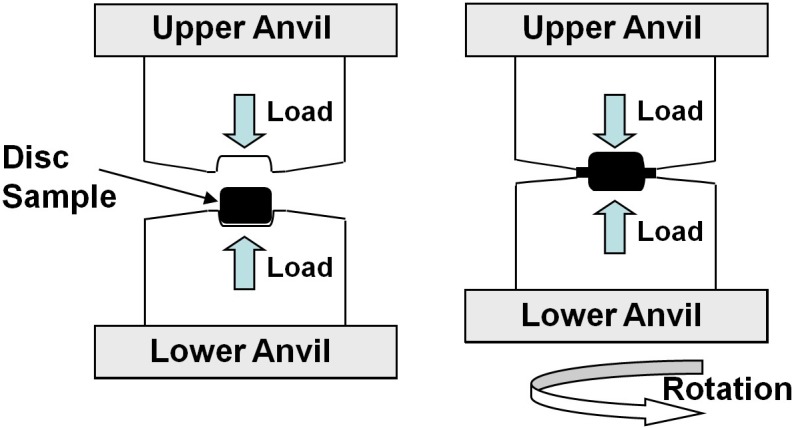
Schematic illustration of high-pressure torsion (HPT) [41] (used with permission from Elsevier).

In the high-pressure torsion (HPT) method, as schematically shown in Figure 1, a small disc sample or small amount of powder is squeezed between two anvils under high pressure and concurrently strained by rotating the lower anvil with respect to the upper anvil [1]. From a historical point of view, the principle of HPT was introduced eight decades ago at Harvard University by Bridgman [2], who mainly used the method to investigate the phase transformations under high pressure. Within the last century, the Bridgman method was used in different science and engineering fields to examine the properties and phase transformations under high pressure (see a review in [3]). In 1988, Valiev et al. successfully employed the HPT technique to achieve significant grain refinement to the submicrometer level in an alloy [4]. Since 1988, the HPT method has been widely used as a severe plastic deformation (SPD) method (see detailed definition of SPD [5]) by different groups to study the microstructure–property relations in ultrafine-grained (UFG) materials [6,7].

In 2007, Japanese researchers employed the HPT method to enhance the hydrogen storage kinetics in an MgNi_2_ intermetallic [8]. This study, together with a few publications on other SPD methods such as equal-channel angular pressing (ECAP) [9–11] and accumulative roll-bonding (ARB) [12–15], reported significant improvement in the hydrogen storage kinetics of Mg-based alloys. Since 2007, there have been numerous successful attempts to improve the hydrogenation kinetics not only by HPT processing [16–21] but also by ECAP [22–28] and ARB [27–32].

Our group widely investigated the effect of HPT processing on hydrogen storage properties of different Mg-based [33–38] and Ti-based materials [39–43]. As will be reviewed in this paper, we employed the HPT method for three main reasons which are of importance in future application of metal hydrides for hydrogen storage (see some reviews on hydrogen storage materials in [44–46]): first, production of bulk UFG hydrogen storage materials to facilitate the activation for hydrogenation and improve the air resistivity to deactivation; second, introduction of different kinds of lattice defects such as grain boundaries, amorphous regions, stacking faults and dislocations to enhance the hydrogen storage performance; and third, synthesis of new hydrogen storage materials.

## 2. Enhancement of activation and air resistivity by HPT

**Figure 2.**
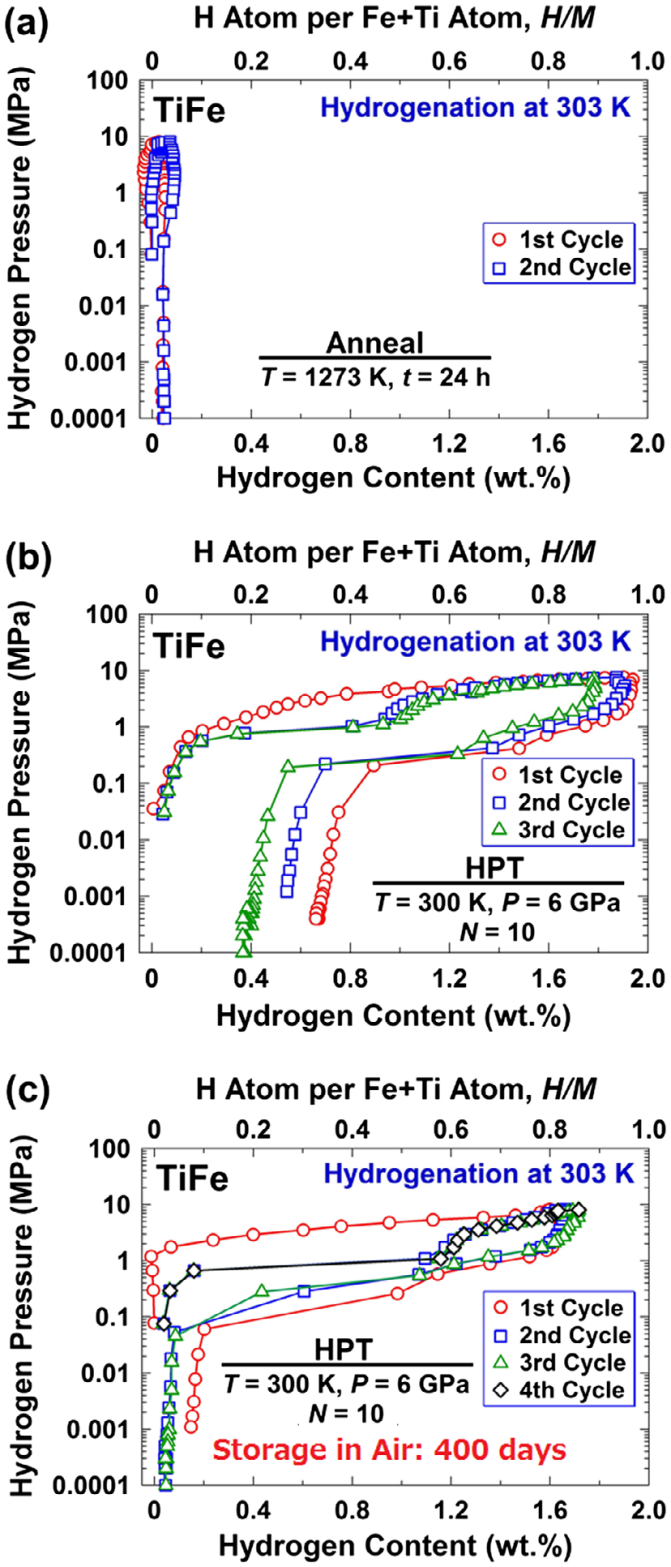
PCI plots at 303 K for TiFe processed by (a) annealing at 1273 K for 24 h [39], (b) HPT for *N* = 10 turns without storage in air [42] and (c) HPT for *N* = 10 turns followed by 400 days storage in air [39] (used with permission from Elsevier).

The TiFe intermetallic was introduced four decades ago as a hydrogen storage material with low price and excellent capability for reversible hydrogen absorption and desorption at room temperature [47]. However, the material did not receive appreciable attention because of two major drawbacks [47]. First it suffers from difficult activation in the first hydrogenation cycle due to the presence of a passive oxide on the surface. Second, the material is deactivated quickly once it is exposed to air. TiFe is usually activated under vacuum or hydrogen atmosphere by increasing the temperature to 673 K [47]. In order to overcome the activation problem of TiFe, the material after ingot casting was processed by HPT [39]. The examination of material by pressure–temperature isotherms (PCI) confirmed that although the as-cast sample was not active for hydrogen storage (Figure 2(a)), the HPT-processed sample absorbed and desorbed hydrogen at ambient temperature without any thermal activation treatment (Figure 2(b)). In an attempt to examine the air resistivity of the HPT-processed sample to deactivation, the material was stored in air atmosphere for 400 days and subsequently examined by PCI analysis [39]. As shown in Figure 2(c), the HPT-activated material readily absorbed hydrogen even after such a long storage in air, confirming that both difficult activation and easy deactivation of TiFe could be overcome by HPT processing.

**Figure 3.**
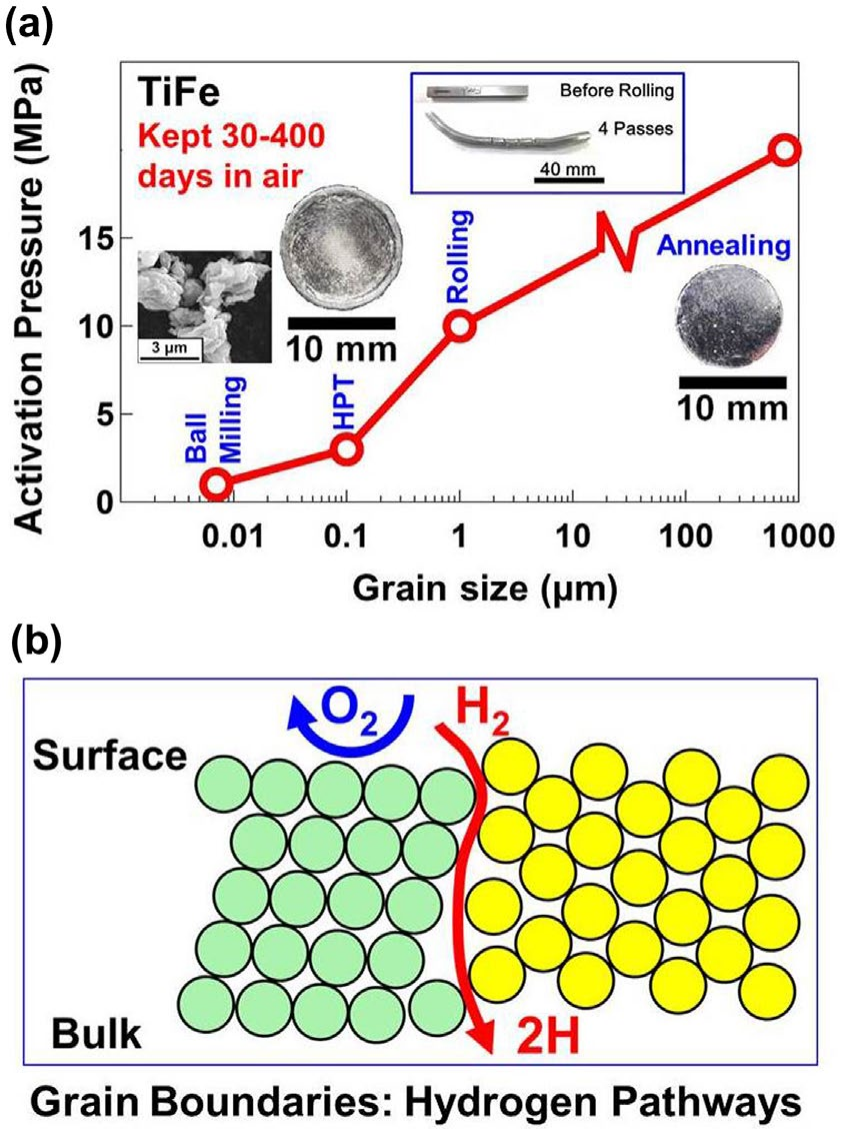
(a) Grain size effect on activation pressure of TiFe processed by annealing [39], groove rolling [41], HPT [39] and ball milling [48] (inset for appearance of samples after processing). (b) Schematic illustration of impact of grain boundaries as hydrogen pathways on easy activation for hydrogenation and difficult deactivation in air [40] (used with permission from Elsevier and AIP).

The mechanism of activation of TiFe by HPT processing was investigated in detail by analyzing the microstructure and surface features using different techniques [40]. It was found that formation of large fraction grain boundaries as hydrogen pathways should be the main reason for easy activation, although the formation of some catalytically active surface segregations and cracks could also partly influence the high performance of the material. To clarify the correlation between the ease of activation and large fraction of grain boundaries, TiFe samples with different grain sizes were produced by four processing routes: coarse grains by thermal annealing at 1273 K [39], micrometer-sized grains by groove rolling [41], submicrometer grains and nanograins by HPT processing [39] and nanograins by high-energy ball milling [48]. As summarized in Figure 3(a), the coarse-grained material could not be activated under low pressures, while the activation pressure was decreased with the reduction of grain size (i.e. by increasing the fraction of high-angle grain boundaries). The lowest activation pressure was achieved after ball milling because the material had the smallest grain size [48]. These experiments indicated that the grain boundaries are effective pathways to transport hydrogen from the surface to the bulk, but they do not act as pathways for oxygen transport to deactivate the material (see Figure 3(b) for the suggested mechanism). Easy activation (i.e. fast hydrogenation kinetics in the first cycle) is not limited to the HPT-processed TiFe intermetallics, as similar results were achieved for pure Mg [33], Mg_2_Ni intermetallics [34], Ti–V alloys [43] and Ti–Fe–Mn intermetallics [42].

## 3. Impact of lattice defects generated by HPT

**Figure 4.**
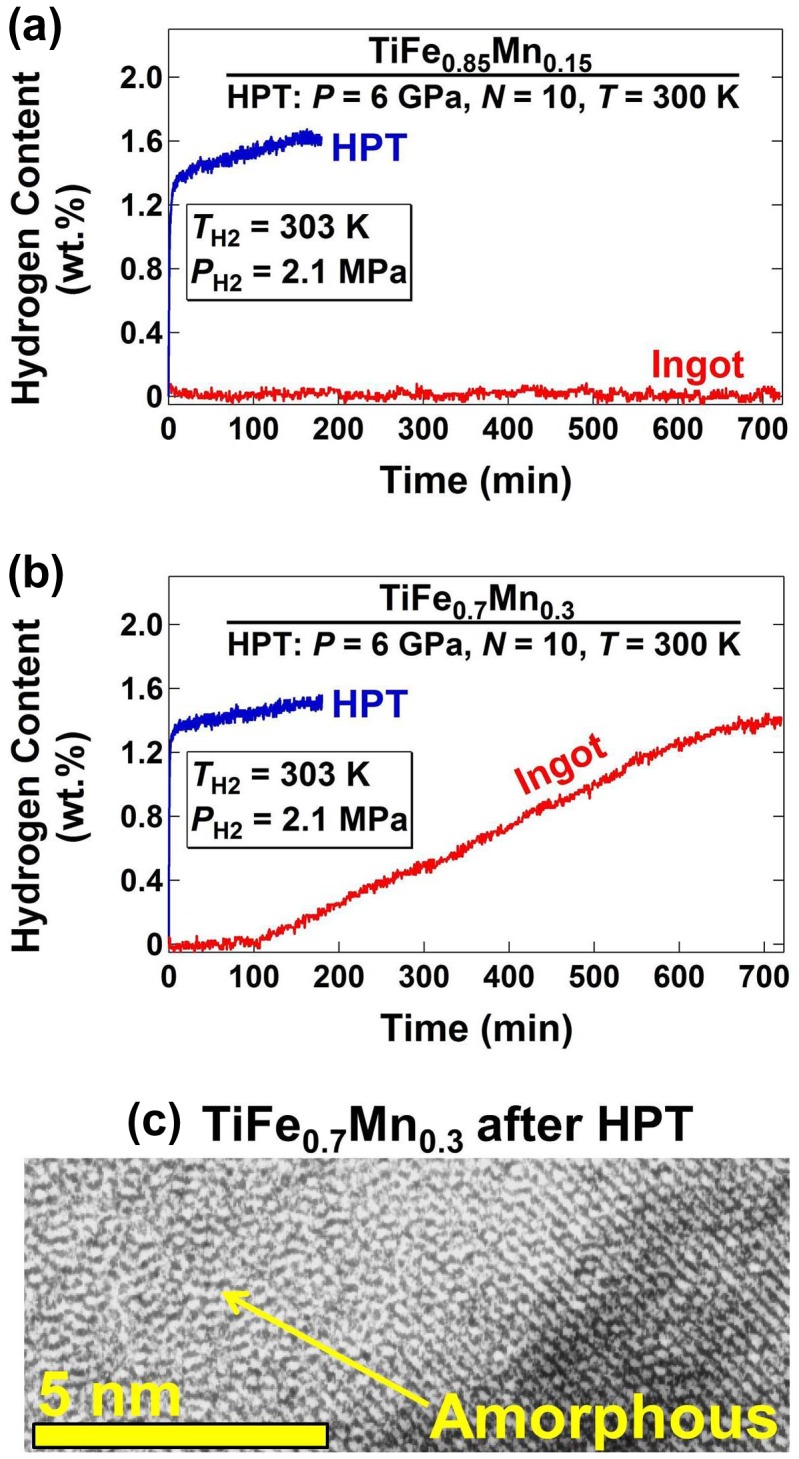
Hydrogen content vs. hydrogenation time at 303 K under initial hydrogen pressure of 2.1 MPa for (a) TiFe_0.85_Mn_0.15_ and (b) TiFe_0.7_Mn_0.3_ intermetallics before (ingot) and after processing by HPT for *N* = 10 turns [42]. (c) High-resolution TEM image of HPT-processed TiFe_0.7_Mn_0.3_ with amorphous region indicated by arrow [42] (used with permission from Elsevier).

Figure 4 shows the hydrogenation kinetic curves in the first hydrogenation cycle for two Ti–Fe–Mn intermetallics before and after HPT processing: (a) TiFe_0.85_Mn_0.15_ and (b) TiFe_0.7_Mn_0.3_. It should be noted that although the addition of Mn to TiFe improves the activation, the materials still needs an activation treatment because of a long incubation period for hydrogen transport thought the oxide layer [49,50]. As shown in Figure 4(a) and (b), while the as-cast intermetallics exhibited slow kinetics at room temperature, the HPT-processed materials absorbed hydrogen very fast without any incubation period [42]. It was found that the improvement of the hydrogenation performance after HPT processing was not only due to the grain refinement but also due to partial formation of defected amorphous regions as pathways for fast hydrogen transport, as shown in the lattice image of Figure 4(c) taken by transmission electron microscopy (TEM) [42].

**Figure 5.**
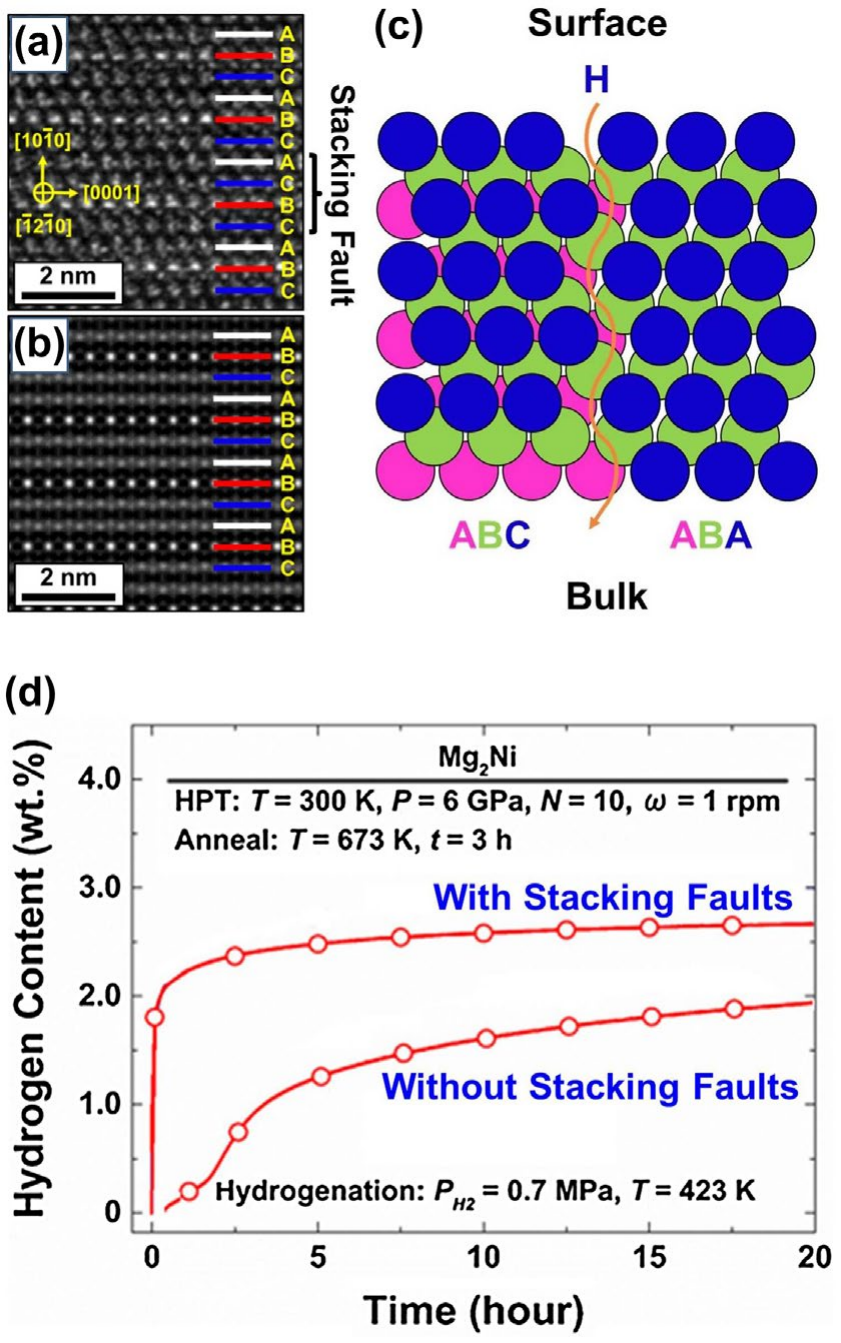
(a) TEM lattice image of stacking faults formed in Mg_2_Ni by HPT processing followed by annealing at 673 K; (b) simulated ideal atomic stacking in Mg_2_Ni; (c) schematic illustration of effect of stacking faults as hydrogen pathways on easy activation and fast hydrogenation kinetics; and (d) hydrogen content vs. time at 423 K under initial hydrogen pressure of 0.7 MPa for Mg_2_Ni after annealing at 673 K with coarse grains (lower curve) and after HPT processing followed by annealing at 673 K with stacking faults in coarse grains (upper curve) [34] (used with permission from Elsevier).

While grain boundaries and amorphous regions have a significant effect on hydrogen storage, other lattice defects produced by HPT processing such as stacking faults and dislocations can also influence the hydrogen storage properties. To study the impact of stacking faults on hydrogen storage performance, an Mg_2_Ni intermetallic was processed by two routes to produce different microstructural features [34]: (1) casting followed by thermal annealing at 673 K to produce coarse grains; and (2) HPT processing followed by annealing at 673 K to produce coarse grains with large fractions of stacking faults (see the formation of an ACBC stacking fault in Figure 5(a) when compared to the ideal ABCABC structure in Figure 5(b)). Basically, stacking faults coupled with partial dislocation can produce atomic pathways which can transport hydrogen, as schematically shown in Figure 5(c). Figure 5(d) shows the hydrogenation kinetic curves for Mg_2_Ni with and without the presence of stacking faults. It is apparent that while the Mg_2_Ni sample with large fractions of stacking faults could absorb 2 wt.% of hydrogen within a few minutes, the coarse grained material could not absorb similar amount of hydrogen even after 20 h. These results confirm that the stacking faults produced by HPT processing can be as effective as grain boundaries to enhance the hydrogenation kinetics and activation [34].

**Figure 6.**
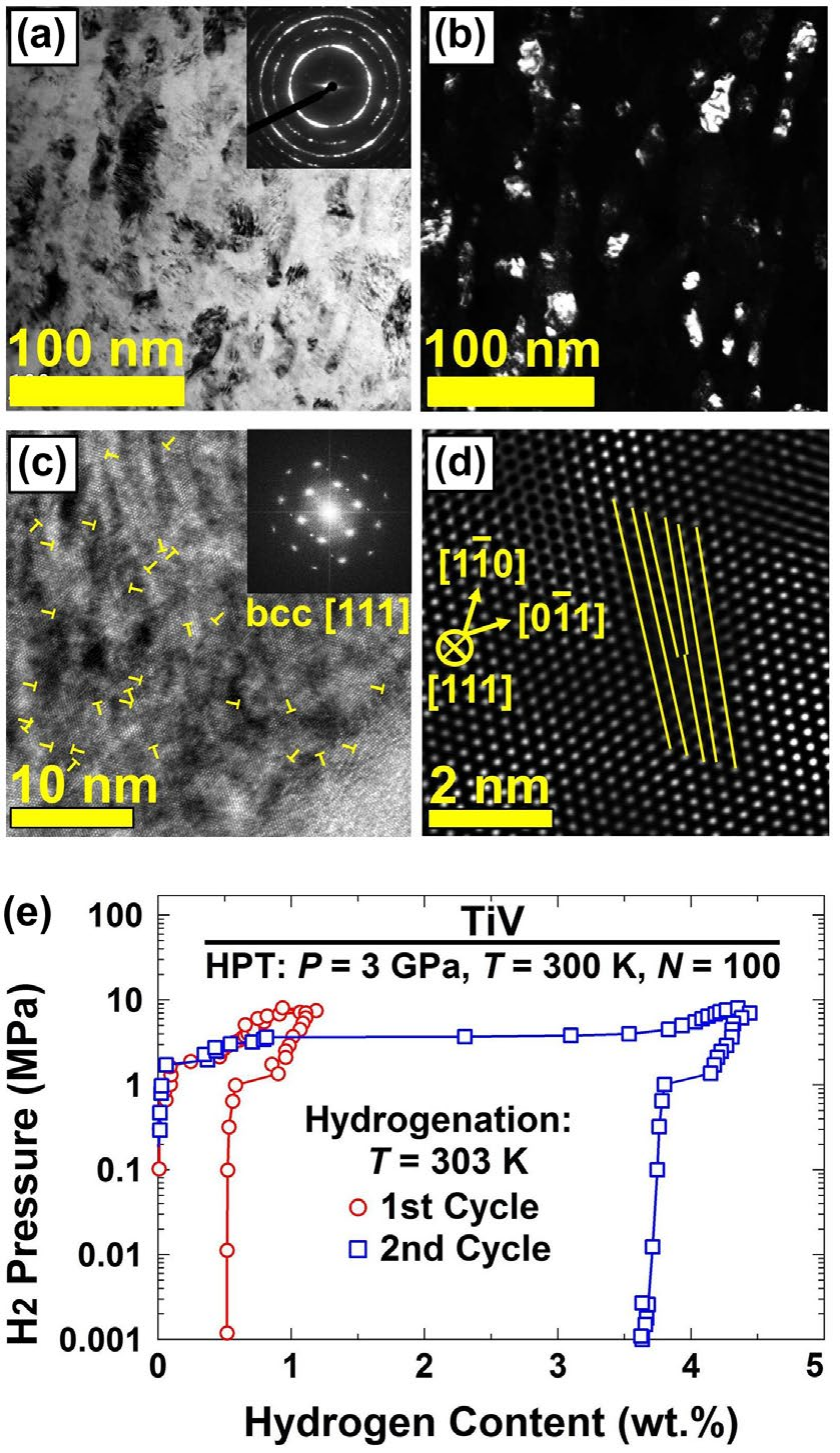
(a) TEM bright-field image with SAED analysis, (b) TEM dark-field image of nanograins, (c) high-resolution image with edge dislocations marked with **T** and corresponding FFT analysis, (d) lattice images of an edge dislocation and (e) PCI plots at 303 K for Ti–V alloy synthesized from Ti and V powders by HPT processing for *N* = 100 turns [43] (used with permission from Elsevier).

Similar to TiFe-based intermetallics, Ti–V-based alloys thermodynamically absorb hydrogen at room temperature [51], but hydrogenation does not occur easily without an activation process because both Ti and V produce passive oxides in air atmosphere [52]. In an attempt to ease the activation of Ti–V alloys, we synthesized a Ti–V alloy from the Ti and V powders using the HPT method [43]. X-ray diffraction (XRD) analysis and elemental mapping using scanning transmission electron microscopy (STEM) confirmed that Ti and V were totally mixed at very large strains. The Ti–V alloy at large strain had a nanograined bcc structure with an average grain size of 35 ± 20 nm, as shown in Figure 6(a) and (b). Moreover both XRD analysis and high-resolution TEM observations confirm the formation of an ultrahigh density of edge dislocations in the range of 10^16^ m^−2^ after HPT processing (see Figure 6(c) and (d)). The presence of large fractions of grain boundaries and edge dislocations activated the material and facilitated the metal-hydride phase transition and as a result, the material absorbed ~4 wt.% of hydrogen at room temperature in the second hydrogenation cycle after an incubation period (see PCI analysis in Figure 6(e)). The kinetic measurements suggested that the hydrogen absorption in the incubation period is controlled by the slow rate of hydrogen dissociation on the surface oxide [43]. It should be noted that although the lattice defects could enhance the activation of Ti–V alloy, the effect of these HPT-induced lattice defects on hydrogen storage reversibility should be examined in future works because it is believed that dislocations have a negative effect on the reversibility of Ti–V-based alloys [53].

## 4. Synthesis of hydrogen storage materials by HPT

Since the HPT method is quite effective to control the solid-state phase transformations [54], to synthesize new alloys [55] and to produce the intermetallics at low temperatures [56], our group, employed this method widely to synthesize different Mg-based and Ti-based hydrogen storage materials. In an attempt to examine the capability of the method to synthesize Mg-based compounds, Mg powders were mechanically mixed with the powders of 21 different elements and the powders mixtures were processed by HPT [36]. Nanograined intermetallics were successfully formed after the HPT process or after short-term post-HPT heat treatment in all of the systems which have stable intermetallic phases [36]. Moreover, metastable or amorphous phases were formed in some of the selected binary systems such as amorphous phases in the Mg–Al [36] and Mg–Zn [36] systems or metastable bcc, fcc and hcp phases in the Mg–Ti [35] and Mg–Zr [37] systems. In another study, it was found that new phases could be synthesized by the HPT method in ternary Mg-based systems such as Mg–V–Sn, Mg–V–Pd, Mg–V–Ni, Mg–Ni–Sn and Mg–Ni–Pd [38]. The HPT method was also effective to synthesize Ti-based materials such as Ti–Al [57], Ti–Nb [58], Ti–V [43] and Ti–Ni–Al [59]. Here, we show three examples on the potential of HPT method to synthesize new Mg-based hydrogen storage materials.

**Figure 7.**
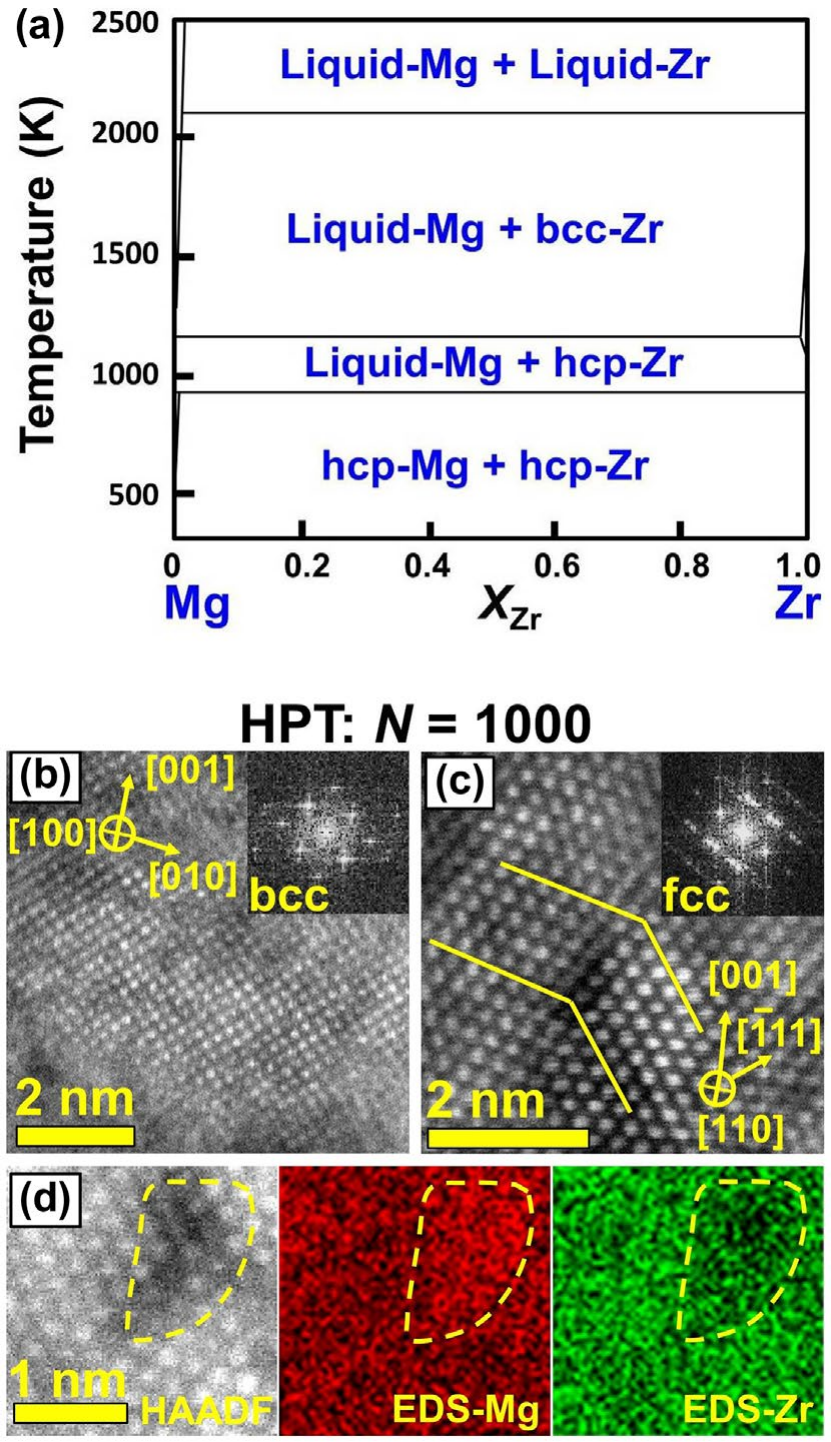
(a) Phase diagram of binary Mg–Zr system [60]. STEM lattice image of (b) bcc phase, (c) fcc phase with nanotwin and (d) Mg nanocluster for Mg–Zr alloy processed by HPT for *N* = 1000 turns, where FFT analyses were included in (b) and (c) and elemental mapping was included in (d) [37] (used with permission from Elsevier).

Mg and Zr with hcp crystal structures are totally immiscible even in the liquid form and at high temperatures, as shown in the binary phase diagram of Figure 7(a) [60]. However, when the Mg–Zr powder mixtures were processed by HPT, the two elements were significantly dissolved in each other [37]. In addition to the formation of supersaturated hcp phase, a new nanostructured bcc phase (Figure 7(b)), a nano-twinned fcc phase (Figure 7(c)) and Mg-based nanoclusters (Figure 7(d)) were formed in the Mg–Zr system [37]. The application of HPT to the immiscible Mg–Ti system also resulted in the formation of supersaturated hcp, bcc and fcc phases [35], in good agreement with earlier results on the application of long-term ball milling to the Mg–Ti powder mixture [61]. Examination of hydrogen storage properties confirmed that while the HPT-processed Mg–Zr absorbed ~1 wt.% of hydrogen under 9 MPa within 20 s and fully desorbed the hydrogen in air atmosphere [37], the Mg–Ti metastable phases could not absorb hydrogen at room temperature [35]. The fast hydrogenation of HPT-processed Mg–Zr was attributed to the absorption of hydrogen in Mg nanoclusters, as shown in Figure 7(d) [20].

**Figure 8.**
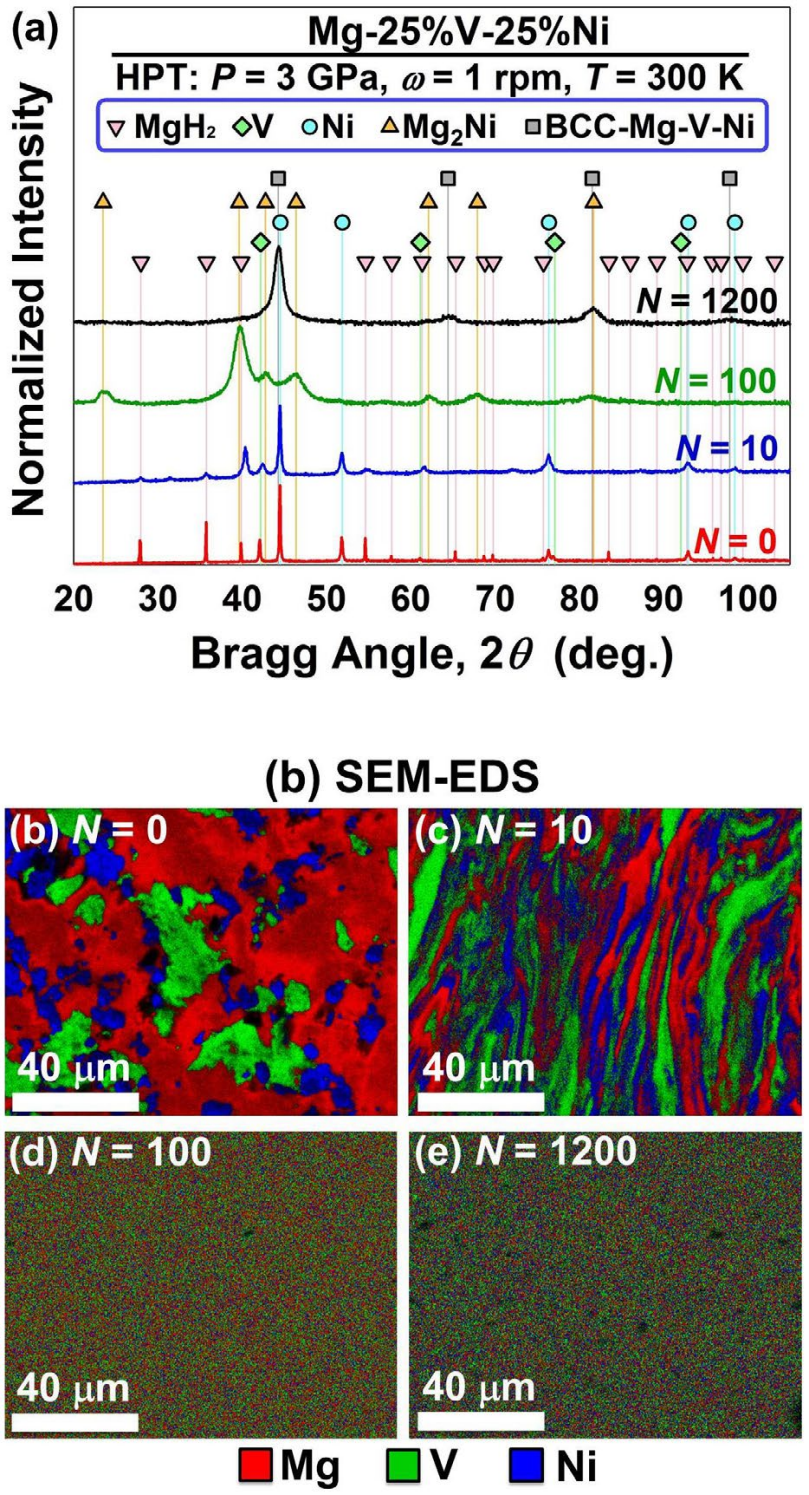
(a) XRD profiles and (b–e) STEM elemental mapping for Mg–V–Ni alloy synthesized from MgH_2_, V and Ni powders by HPT processing for *N* = 0 (only compression), 10, 100 and 1200 [38] (used with permission from Elsevier).

The HPT method can be employed to synthesize not only the binary alloys but also new ternary Mg-based alloys [38]. As shown in the XRD pattern of Figure 8(a), when powder mixtures of MgH_2_, V and Ni were processed by HPT, a pure bcc phase with a composition of Mg_2_VNi was formed [38]. Elemental mapping by scanning electron microscopy (SEM), as shown in Figure 8(b)–(e), also confirm that the three elements were mixed uniformly by increasing the number of HPT turns and a complete homogeneity was achieved after *N* = 1200. The formation of Mg_2_VNi with the bcc structure is comparable with an earlier report on long-term ball milling of this system which could not produce a single bcc phase in the Mg_2_VNi composition [62].

**Figure 9.**
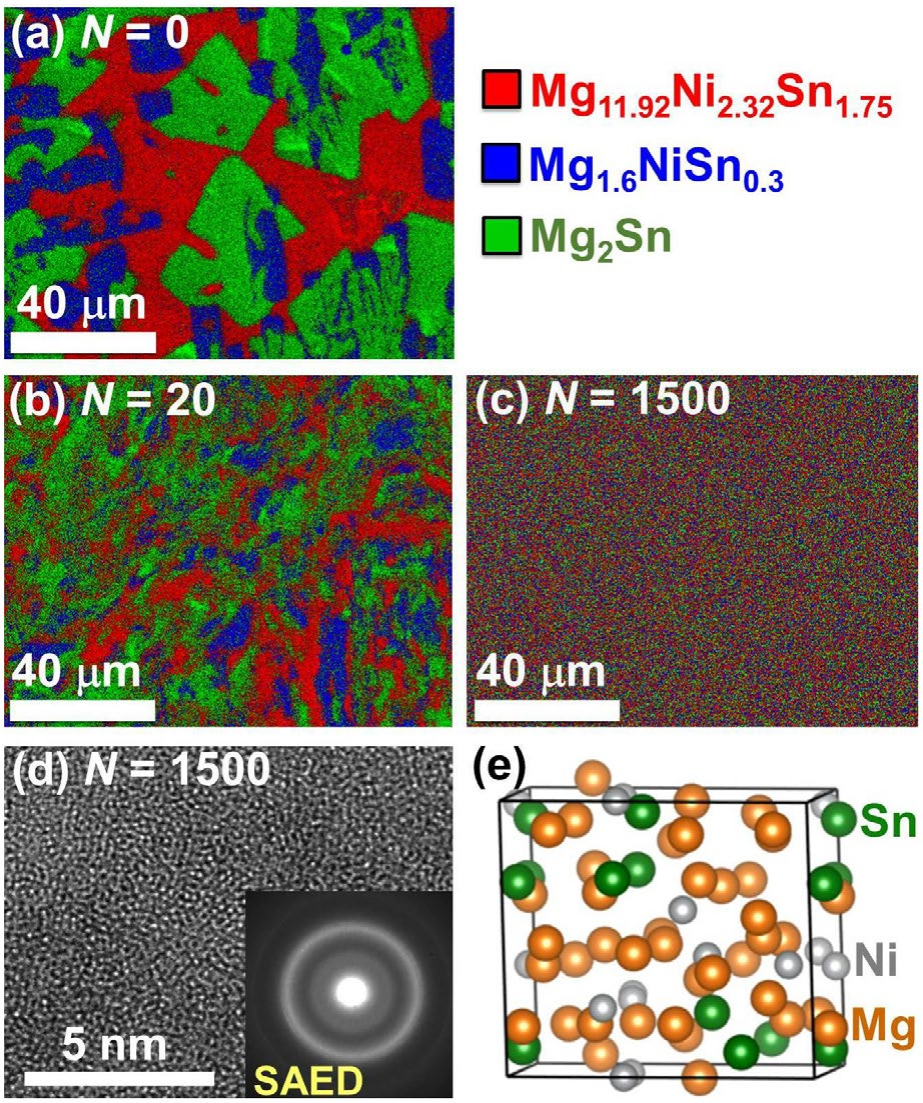
(a–c) STEM elemental mapping, (d) TEM high-resolution image and SAED analysis and (e) optimized amorphous structure achieved by first-principles calculations for Mg–Ni–Sn alloy processed by HPT for (a) *N* = 0 (as-received ingot), (b) *N* = 20 and (c, d) *N* = 1500 [38] (used with permission from Elsevier).

Figure 9 shows another example on the potential of the HPT method to synthesize new ternary Mg-based hydrogen storage materials with an amorphous structure [38]. An ingot of Mg_4_NiSn which contained three intermetallics (Figure 9(a)) was processed by HPT. While the material processed for *N* = 20 turns contained the initial intermetallics in a fragmented form (Figure 9(b)), the material after ultra-SPD for *N* = 1500 HPT turns exhibited a uniform mixture of the three elements (Figure 9(c)). The XRD analysis as well as transmission electron microscopy confirmed that the synthesized material had an amorphous structure [38], as evident from the lattice image of Figure 9(d) and its corresponding selected area electron diffraction (SAED) with a hollow ring pattern. First-principles calculations, as shown in Figure 9(e), also confirmed that the atomic-scale mixing of Mg, Ni and Sn should result in amorphization of the structure in good agreement with the experimental results [38]. Although the HPT method is quite effective to synthesize new hydrogen storage materials, the number of HPT turns (i.e. shear strain) should be in the range of *N* = 100–1000 which is much higher than those usually required to achieve mere grain refinement or maximum hardening in metals [63], semi-metals [64], alloys [65] and ceramics [66] (usually *N* = 1–10).

## 5. Concluding remarks

In conclusion, the HPT process provides new opportunities to activate hydrogen storage materials, to improve their resistance to deactivation in the air, to enhance their hydrogenation kinetics as well as to synthesize new hydrogen storage materials with unique compositional and microstructural features. All these features are due to the effect of HPT processing on the introduction of lattice defects including the grain boundaries. The lattice defects can enhance the diffusion, act as pathways for hydrogen transport and ease the phase transformations and accelerate mechanical alloying. These features are similar to those reported on the potential of ball milling in enhancement of hydrogenation kinetics [67–69], ease of activation [70–72] and synthesis and tailoring of hydrogen storage materials [73–75]. However, there are two main differences between these two methods. First, the material is deformed by pure shear deformation in HPT processing which makes the method appropriate for fundamental studies, but the material in ball milling experiences complicated deformation process with some contamination from the balls and vial. Second, the sample after HPT processing is in the bulk form with high resistance to air atmosphere, but the sample in ball milling is in the form of powder with very high surface activity and should be usually stored under a controlled atmosphere.

## Disclosure statement

No potential conflict of interest was reported by the authors.

## Funding

This work was supported by the MEXT, Japan for a Grant-in-Aid for Scientific Research (B) [number 16H04539] and Grant-in-Aid for Scientific Research (S) [number 26220909].
